# NLRP3 inflammasomes at the ARDS-cancer interface: mechanisms and translational hypotheses

**DOI:** 10.3389/fimmu.2026.1798542

**Published:** 2026-05-05

**Authors:** Salima Shebbo, Doaa Elsayed Mahmoud, Nooralhuda Alateyah, Seyedeh H. Hosseini, Amal Al-Haidose, Salam A. Ramirez, Asad Zeidan, Atiyeh M. Abdallah

**Affiliations:** 1 Department of Biomedical Science, College of Health Sciences, Qatar University (QU) Health, Qatar University, Doha, Qatar; 2 Department of Basic Medical Sciences, College of Medicine, Qatar University (QU) Health, Qatar University, Doha, Qatar; 3 College of Dental Medicine, Qatar University (QU) Health, Qatar University, Doha, Qatar; 4 Department of Pharmaceutical Sciences, College of Pharmacy, Qatar University (QU) Health, Qatar University, Doha, Qatar; 5 Department of Chemistry, Royal College of Surgeons in Ireland, University of Medicine and Health Sciences, Dublin, Ireland

**Keywords:** acute respiratory distress syndrome, cancer, chronic inflammation, immune dysregulation, inflammasomes

## Abstract

Acute respiratory distress syndrome (ARDS) and cancer share common pathogenetic mechanisms that involve immune dysregulation and inflammatory responses. Inflammasomes, part of the initial innate immune response to infection and injury, play a pivotal role in both carcinogenesis and ARDS. Here we review the role of inflammasomes in ARDS and cancer and, in doing so, we find a set of commonalities that allow the proposal of a conceptual framework for their potential intersection that could inform therapeutic development for both conditions. We highlight the importance of understanding how chronic inflammation induced by ARDS may create a microenvironment conducive to carcinogenesis and discuss how cancer-related inflammasome activity and therapies, like radiotherapy and immunotherapy, might increase susceptibility to ARDS. This review paves the way for future work to investigate strategies to prevent ARDS development in cancer and to repurpose FDA-approved drugs for these diseases, alongside preventive measures to reduce the risk of one disease in the presence of the other.

## 1 Introduction

Acute respiratory distress syndrome (ARDS) is a life-threatening, and in some cases fatal, respiratory disease that develops due to overly responsive or inadequately controlled inflammatory reactions in the lungs, and it develops in about one in ten of all ICU admissions (1–3). First described by Ashbaugh and colleagues in 1967, ARDS remains a clinical challenge, and there are still no FDA-approved pharmacological therapies for the condition. As a result, progress in improving clinical outcomes for patients with ARDS has been limited (4). Two fifths of patients who develop ARDS die from their disease (5). At the same time, the burden of cancer has continued to increase, with ~20 million new cases and 9.7 million deaths worldwide in 2022 (6). Although there have been significant advances in cancer therapy, including the development of immunotherapy, relatively few patients with cancer can be considered “cured”, with cancer now managed as a chronic disease over the long term in many cases (7).

Neutrophils are innate immune system cells and the principal cell type driving ARDS following direct or indirect lung injury. ARDS can be classified into ‘hypo-’ or ‘hyper-inflammatory’ disease, the latter accounting for ~30% of cases and associated with higher mortality rates (8–10). In ARDS, and especially hyper-inflammatory ARDS, neutrophil activation leads to the secretion of the proinflammatory cytokines interleukin (IL)-1 and IL-18 via pathways related to inflammasomes (4, 11), multiprotein signaling hubs that play a crucial role in controlling host defense mechanisms and inflammatory responses (11). After detecting danger signals from pattern-recognition receptors (PRRs) on neutrophils, inflammasomes activate inflammatory caspases, cytokine production, and an inflammatory form of programmed cell death known as pyroptosis. In ARDS, inflammasomes are usually activated by pathogen-associated (PAMP) or damage-associated (DAMP) molecular patterns, which trigger common downstream effectors related to ARDS development. Inflammasomes contribute to ARDS development though activation of several pathways related to PRRs and inflammasomes such as NLRP3, NLRC4, AIM2, and pyrin (4, 12).

Furthermore, inflammasomes, especially AIM2, NLRP6, NLRC4, NLRP3, and NLRP1, play a pivotal role in cancer by modulating adaptive and innate immune responses and influencing key cancer hallmarks like cell proliferation and death (13). Additionally, ARDS and cancer share common inflammatory pathways, particularly those related to inflammasomes. For instance, inflammasomes have been reported to be activated in response to metabolic dysregulation and oncogenic stress, and NLRP3 inflammasomes are thought to determine the trajectory of tumor cell fate (14). Given this commonality, here we review the mechanisms by which inflammasomes might contribute to the development of ARDS and cancer. In doing so, we devise a hypothetical framework for a bidirectional relationship between cancer and ARDS development and their mutual contribution to disease progression, concentrating particularly on the role of NLRP3 inflammasomes in this dynamic. We hypothesize that inflammasome-mediated inflammation provides a bidirectional link between ARDS and cancer. According to this hypothesis, acute and chronic sequelae of ARDS may promote tumor initiation and progression, while cancer and its therapies can precipitate ARDS via the same pathways. In writing this review, we aim to highlight a potentially rewarding but neglected research area that warrants further systematic study, especially the need for well-designed epidemiological studies and Mendelian randomization analyses to clarify and potentially prove the association, especially regarding the progression from ARDS to cancer.

## 2 Inflammasomes: a brief overview

Inflammasomes are multi-protein complexes that form part of the innate immune system (15, 16). They are broadly classified into classical and non-classical types, depending on the set of proteins activated. Upon sensing PAMPs or DAMPs, inflammasomes recruit the adaptor protein ASC and activate caspase-1. Caspase-1 then drives two key processes: cleavage of pro-IL-1β and pro-IL-18 into their mature, secreted forms, and cleavage of gasdermin-D to induce pyroptosis, a highly inflammatory form of cell death (15). Classical inflammasomes are multiprotein complexes that contain one of three NOD-like receptors (NLRs) (NLRC4, NLRP3, and NLRP1) or two non-NLR proteins (pyrin and proteins absent in melanoma (AIM) 2) (11). Since then, several more inflammasome-related sensor proteins have been discovered, including NLRP12 (17), NLRP11, NLRP10, NLRP9, NLRP7, NLRP6, MxA, and CARD8 (18). Also, IFI16 was found to interact with apoptosis-associated speck-like protein (ASC) through a pyrin domain (PYD) and create IFI16-ASC inflammasomes (19). These sensors are capable of perceiving various bacterial, viral, or protozoal PAMPs. They can also sense reactive oxygen species (ROS)-, mitochondrial damage-, stress-, and heme-induced DAMPs (18).

## 3 Acute respiratory distress syndrome

### 3.1 Pathophysiology

ARDS is characterized by diffuse alveolar damage, capillary leak, severe hypoxemia, and an overwhelming cytokine storm. Despite decades of intensive research efforts, the pathogenesis of ARDS is still not completely understood. ARDS arises through the initiation and dysregulation of several interacting injury response pathways, inflammation, and both pulmonary and systemic coagulation defects (20). In ARDS, both layers of the alveolar–capillary barrier, formed from closely apposed alveolar epithelial cells and capillary endothelial cells separated by a basement membrane that helps gas exchange, are damaged. Injury to this barrier therefore adversely affects gas exchange and work of breathing in individuals with ARDS. The increased alveolar–capillary barrier permeability results in alveolar edema, which is exaggerated by a breakdown in normal transport mechanisms for alveolar epithelial fluid, which usually exports fluid into the interstitium for clearance via the circulation and lymphatics. Alveolar flooding has major consequences in terms of impaired gas exchange, surfactant inactivation, decreased lung compliance, and a consequent increase in work of breathing (21). Furthermore, activation of procoagulant pathways in the lung endothelium causes lung microvascular thromboses, which increase dead space, in turn contributing to significantly impaired gas exchange. Microvascular thromboses and microvascular bed damage trigger pulmonary arterial hypertension and acute ventricular dysfunction, both of which contribute to adverse clinical outcomes (22).

In addition to alveolar-endothelial barrier permeability defects, dysregulated pulmonary inflammatory responses are a hallmark of ARDS. The binding of microbial products or endogenous products of cellular injury to receptors present on the lung epithelium and alveolar macrophages activate innate immune pathways. This results in activation of intrinsic defense mechanisms like macrophage activation, neutrophil recruitment, histone release, and neutrophil extracellular trap (NET) formation, which while helping to capture pathogens, aggravate alveolar injury. NLRP3 inflammasome activation in macrophages and neutrophils initiates local release of IL-1β and IL-18 and subsequent pyroptosis (23). Furthermore, macrophages are activated into a proinflammatory phenotype through PRRs, which bind DAMPs or PAMPs to cause release of proinflammatory cytokines and neutrophil chemoattractants such as IL-8. As a result, neutrophils translocate through the capillary wall via a pathway regulated by interstitial fibroblasts (21).

**Figure 1:**
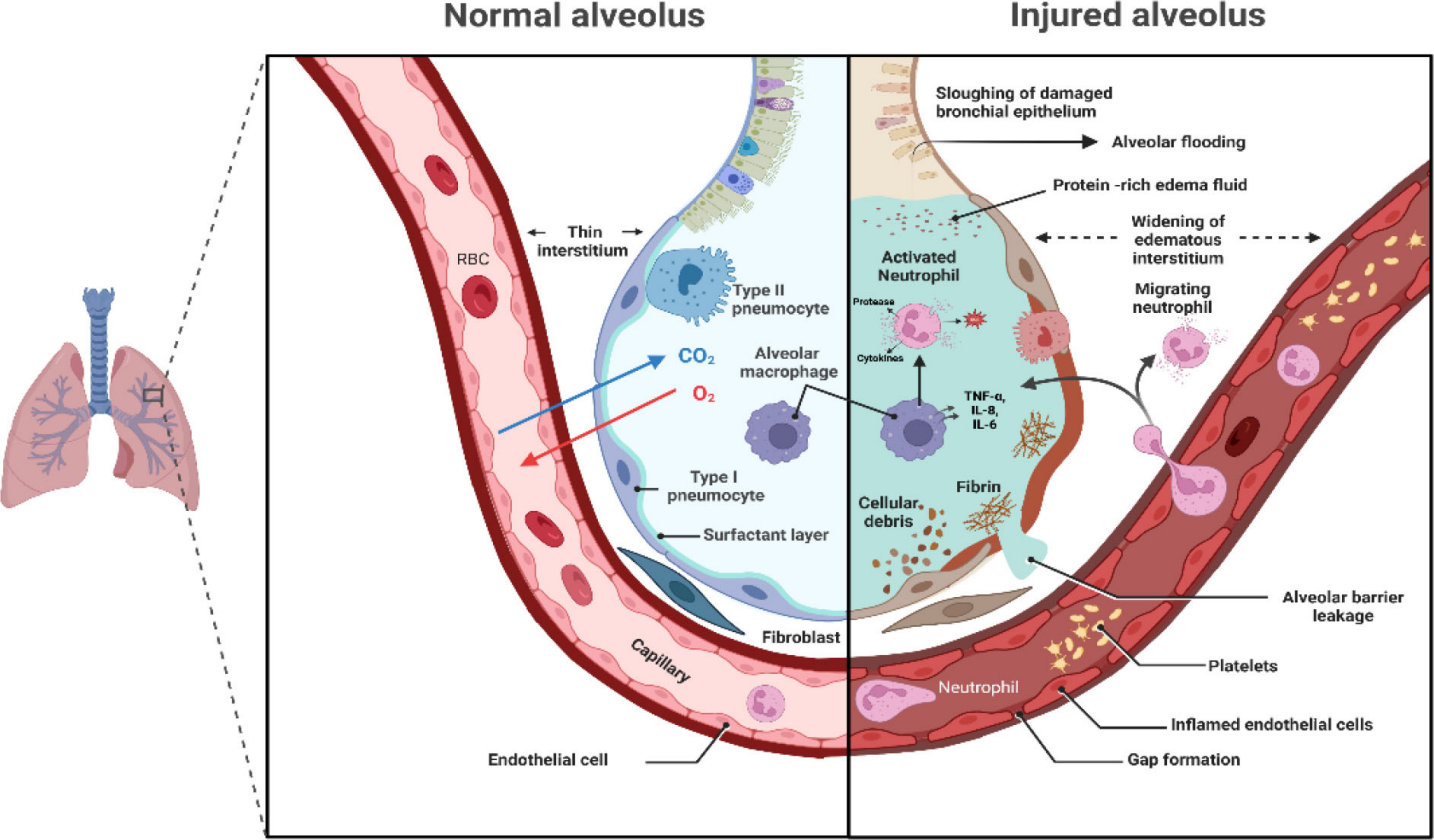
Pathophysiological changes in the alveolus in ARDS. Figure created using BioRender.

A normal and effective response to pulmonary infection and injury therefore relies on a precise balance between a sufficient immune response to resolve the infection or injury and excessive or dysregulated activation that may harm the alveoli (24). In ARDS, this balance is lost, and exaggerated epithelial and endothelial injury in the lung leads to severe local and systemic acute inflammation. **Figure 1** illustrates the pathophysiological changes occurring in the alveolus in ARDS.

### 3.2 Triggers and risk factors for ARDS

#### 3.2.1 Direct and indirect triggers of ARDS

The most common trigger for ARDS, accounting for ~40% of cases, is sepsis. Sepsis of pulmonary origin is more likely to cause ARDS through both direct and indirect ALI, where ARDS represents the most severe clinical manifestation of ALI (25). Gram-positive and Gram-negative bacterial pneumonia is another common cause of ARDS, especially in hospitalized patients with a positive microbiological diagnosis. While viral and fungal pathogens are less frequent causes of pneumonia, they are associated with higher risk of ARDS than bacterial pneumonia. Aspiration of gastric contents accounts for ~30% of ARDS cases and typically results in a more severe form of ARDS, associated with high mortality rates (26). Severe trauma accounts for ~25% of ARDS cases, and these patients have higher survival rates than those with other causes of ARDS. Multiple blood transfusions are also a dose-dependent risk factor for ARDS, with patients receiving fresh frozen plasma being more likely to develop ARDS than those given packed red blood cells (27).

#### 3.2.2 Predisposing risk factors

In addition to these direct triggers, several host-related factors increase susceptibility to ARDS. One notable example is vitamin D deficiency, as vitamin D modulates innate and adaptive immune networks and its deficiency is associated with increased risk of pneumonia and sepsis (28). Indeed, Dancer et al. (28) reported that all ARDS patients and about 96% of un-supplemented esophagectomy patients have vitamin D deficiency compared to controls. Patients with severe vitamin D deficiency express increased levels of alveolar damage markers, which were suppressed in patients receiving vitamin D_3_ supplementation prior to esophagectomy. Therefore, vitamin D supplements may have a therapeutic and preventive role in ARDS.

### 3.3 Key molecular pathways in ARDS

In lung cells, several molecular pathways are implicated in the pathogenesis of ARDS, particularly inflammasome, nuclear factor-kappa B (NF-κB), oxidative stress, apoptotic and cell death, and complement pathways.

#### 3.3.1 NF-κB pathway

The NF-κB pathway, initially discovered in B cells, has since been identified as active in various other cell types such as endothelial and innate immune cells. Given its essential role in controlling the development and resolution of inflammation, NF-κB signaling is deemed a major inflammatory regulator (29–31). Sustained activation of NF-κB signaling eventually leads to lung injury and fibrosis (32, 33). In the context of ARDS, NF-κB is primarily associated with pneumonia- or sepsis-mediated ARDS, as NF-κB is a primary downstream effector after DAMP or PAMP recognition by PRRs expressed by host cells in response to cell death, stress, or injury (34). This leads to the activation of inflammatory pathways involving toll-like receptors (TLRs) which, in turn, trigger activation of NF-κB transcription factors and pro-inflammatory cytokine production (5, 34).

#### 3.3.2 Cell death and apoptosis pathways

Alveolar capillary disruption is intimately linked with endothelial and epithelial cell apoptosis in ARDS (34). This process involves both extrinsic (receptor-mediated) and intrinsic (mitochondria-mediated) pathways. The extrinsic pathway is activated by tumor necrosis factor (TNF) family members like Fas ligand (FasL), which activates caspase-8 through binding to the Fas receptor. Physical and chemical injury tends to activate the intrinsic pathway, resulting in activation of caspase-9 secondary to the release of pro-apoptotic mitochondrial factors like Bak, Bad, and Bax, which localize to and alter the permeability of the outer mitochondrial membrane (35).

#### 3.3.3 Oxidative and endoplasmic reticulum stress pathways

Inflammatory stimuli promote the development of ROS in different cell types, especially recruited leukocytes and alveolar macrophages. ROS induce oxidation and crosslinking of carbohydrates, DNA, lipids, and proteins, eventually leading to cell injury (36). ROS also propagate disruption of the endothelial barrier and hence increase migration of inflammatory cells through the endothelial barrier to aggravate inflammation (34, 37). Several conditions induce ER stress, including viral infections, ischemia, trauma, and sepsis, due to various misfolded or unfolded proteins accumulating in the ER lumen (38). In ER stress pathways, imbalances in cellular homeostasis activate the unfolded protein response (UPR) in response to activating transcription factor 6 (ATF6), inositol-requiring kinase 1α (IRE1α), and protein kinase RNA-like ER kinase (PERK). The UPR usually protects the cell by fragmenting misfolded or unfolded proteins (39), but in the event of ER stress, initiation of proinflammatory NF-κB and MAPK signaling eventually promote lung injury (40–42). However, this pathway may be a potential therapeutic target in ARDS (34).

#### 3.3.4 Role of inflammasomes in ARDS

The role of inflammasomes in ARDS is complex but can be categorized into those related to infective pulmonary causes, sterile pulmonary causes, extra-pulmonary infective causes, and extra-pulmonary sterile causes. In the infective pulmonary causes, pneumonia, caused by viral and bacterial infections, is the leading cause of ARDS (43). In viral and bacterial pneumonia-induced lung injury, neutrophils act as the primary source of IL-1 from both inflammasome-independent and dependent mechanisms. The main inflammasomes involved in pneumonia-induced bacterial infection (e.g., caused by *Streptococcus pneumonia*, *Staphylococcus aureus*, *Pseudomonas aeruginosa*, *Klebsiella pneumonia*, *Burkholderia pseudomallei*, and *Legionella*) are NLRP3 and NLRC4 (44–48). Pneumonia caused by viral infections such as influenza, Middle Eastern respiratory syndrome, and SARS-CoV-1 and 2 have also been reported to be associated with inflammasome activation (e.g., NLRP3). For instance, neutrophil inflammasome activation in SARS-CoV-2 was associated with pyroptosis, formation of apoptosis-associated speck-like protein containing a CARD (ASC speck), production of IL-1β and IL-18, and worse disease severity (49–51). In contrast, the specific inflammasomes involved in sterile (non-infectious) pulmonary injury (e.g., acid-induced lung injury and ventilator-associated lung injury) remains uncertain (4, 52, 53). Extra-pulmonary infective causes mainly include sepsis-induced lung injury, with ~6% of sepsis patients developing ARDS. NLRP3 (the most studied), NLRC4, and AIM2 are all involved in this type of ARDS, and inhibition of NLRP3, along with GSDMD and thioredoxin-interacting protein (TXNIP), protects against lung injury in mouse models of sepsis-induced ALI (54). Also, NLRC4 is upregulated in patients with sepsis, with its levels associated with increased mortality risk (55). Conversely, AIM2 inflammasomes have been shown to be protective in the early immune response to infection in mice, and its inhibition correlated with poorer outcomes (56).

**Figure 2:**
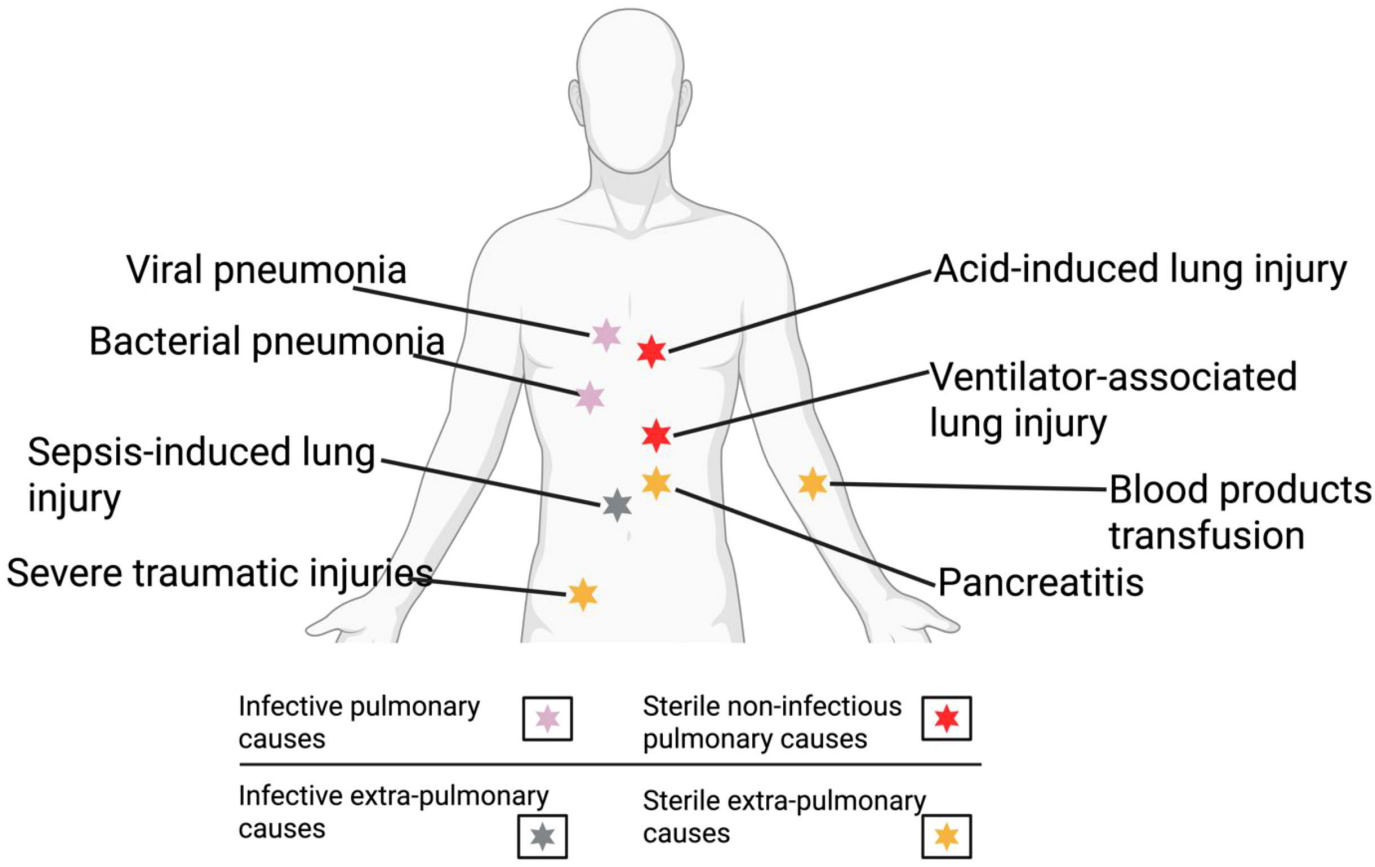
Schematic of the pulmonary and extrapulmonary infective and sterile causes of ARDS. Figure created using BioRender.

ARDS can also be triggered by extra-pulmonary sterile causes, which include massive transfusions of blood products, burns, pancreatitis, and major trauma, all of which injure the lung by initiating inflammatory responses that activate neutrophils (57). An estimated 35-40% of patients with burns who are mechanically ventilated developed ARDS (58). Severe burns can lead to tissue damage, upregulated NLRP3, IL-18, and IL-1β, and increased caspase-1 cleavage (59). Moreover, Roth and co-workers illustrated that AIM2 inflammasomes caused immunosuppression following burn injuries in both humans and mice (60). In severe pancreatitis, release of digestive enzymes damages cells and produces DAMPs, which activate inflammasomes and promote transcription of IL-1β. Sendler et al. investigated the mechanisms by which acute pancreatitis prompts systemic inflammatory response syndrome, inferring that activation of the adaptive immune system results in caspase 1-dependent processes, macrophage-derived cytokine release, and NLRP3 activation (61). There have been fewer studies on NLRC4, so its role in lung injury remains inconclusive (4, 62). Additionally, ARDS develops in up to 25% of polytrauma patients (63), and major trauma such as severe traumatic brain injury (TBI) increases NLRP3 inflammasome assembly, caspase-1 and ASC expression, and IL-1β and IL-18 release (64). **Figure 2** summarizes the pulmonary and extrapulmonary infective and sterile causes of ARDS.

## 4 Cancer and inflammation

### 4.1 Cancer as an inflammatory disease

Chronic inflammation, whether driven by infection, autoimmune disease, or environmental factors, creates a pro-oncogenic microenvironment, and it is recognized as a key hallmark of cancer (65). Several molecular and cellular mechanisms underpin the relationship between inflammation and cancer, shaping the tumor microenvironment (TME) and influencing therapeutic outcomes. Inflammatory cells within the TME, including macrophages, neutrophils, and lymphocytes, release cytokines, chemokines, and growth factors that promote tumor cell survival, angiogenesis, and immune evasion (66).

#### 4.1.1 Hallmarks of cancer associated with inflammation

Tabei and Nakajima recently reported that inflammation-related signaling pathways, particularly those triggered by the proinflammatory cytokine IL-1β, contribute to both inflammatory conditions and cancer by inducing partial epithelial-mesenchymal transition (EMT), a well-established concept in cancer progression whereby epithelial cells lose their normal characteristics and acquire mesenchymal-like motility. IL-1β stimulates two key pathways: the epidermal growth factor receptor (EGFR)-dependent PI3K/AKT pathway and the IL-1R-dependent MEK/ERK pathway. In the first, IL-1β induces activation of matrix metalloproteinases (MMPs) and ADAMs (a disintegrin and metalloproteinase), which shed EGFR ligands such as epidermal growth factor (EGF) and amphiregulin (AREG), leading to EGFR transactivation and downstream PI3K/AKT signaling, ultimately promoting cell survival, motility, and partial EMT. Simultaneously, IL-1β directly activates its receptor (IL-1R), which triggers the IKKβ/Tpl2 cascade [IKKβ (inhibitor of κB kinase β) and Tpl2 (tumor progression locus 2)], finally leading to MEK/ERK activation. Each of these two pathways can, on their own, alter cell behavior related to EMT, like reducing the expression of E-cadherin (an epithelial marker) and increasing levels of vimentin (a mesenchymal marker). However, neither pathway alone can fully trigger the partial EMT state. Instead, both pathways must be activated together, in a coordinated manner, for cells to fully enter this partial EMT condition. This EMT state is crucial, because it is associated with a higher risk of metastasis, resistance to therapy, and tissue fibrosis (65, 67, 68).

Inflammation creates genetic instability in tumors by promoting ROS production and DNA damage. Inflammatory cytokines such as IL-6 and TNF-α also stimulate oncogenic pathways such as NF-κB and STAT3 signaling, enhancing malignant transformation. Given the intricate link between chronic inflammation and cancer, targeting inflammatory pathways has emerged as a promising therapeutic strategy for cancer (69).

#### 4.1.2 Inflammation in tumor initiation

Chronic inflammation plays a crucial role in the early stages of cancer development by damaging DNA, causing epigenetic changes, and creating a supportive environment for the first abnormal cells. Inflammatory immune cells such as macrophages and neutrophils release reactive oxygen and nitrogen species (RONS), which can directly mutate genes like *Tp53* or activate cancer-promoting genes. The persistent presence of these factors increases the likelihood of DNA mutations and genomic instability and ultimately results in malignant transformation. At the same time, inflammatory cytokines like IL-1β, IL-6, and TNF-α change the activity of gene-regulating systems, including DNA methyltransferases (e.g., Dnmt1/3), histone modifiers (e.g., DOTL1), and various microRNAs and long non-coding (lnc)RNA modulators, leading to activation of oncogenic pathways and silencing of tumor suppressor genes. Inflammation also helps normal epithelial cells transform into stem-like tumor-initiating cells, mainly through activation of NF-κB and STAT3 signaling. In organs like the colon, inflammation weakens the epithelial barrier, allowing bacteria like *E. coli* to release genotoxic substances (e.g., colibactin) that contribute to DNA damage. Additionally, chronic inflammation promotes repeated tissue injury, which creates a niche where early tumor cells can survive and proliferate in response to inflammatory signals that foster cell survival, such as STAT3 and NF-κB (66, 68, 70).

#### 4.1.3 Inflammation and metastasis

Chronic inflammation helps cancers spread by initiating EMT, which enhances cancer cell motility and invasion into adjacent tissues. EMT, a process by which epithelial cells become de-polarized and lose adhesion and gain mesenchymal characteristics, is induced by inflammatory cytokines such as TGF-β, IL-6, and TNF-α. These cytokines activate transcription factors, namely Snail, Twist, and Zeb, which suppress epithelial markers such as E-cadherin and activate the expression of mesenchymal markers such as vimentin and N-cadherin. This mechanism enhances the invasion and migration of cancer cells and promotes metastasis from the primary tumor site (71). In addition, NF-κB signaling is an important inflammatory pathway involved in metastasis, with pro-inflammatory cytokines and chemokines such as C-C motif chemokine ligand 2 (CCL2) and C-X-C motif chemokine ligand 8 (CXCL8) recruiting immune cells that secrete MMPs, which degrade the extracellular matrix and permit metastasis. MMP-2 and MMP-9 specifically degrade collagen and other structural components of the extracellular matrix to enable cancer cells to invade nearby tissues and access the blood circulation (72). Moreover, chronic inflammation makes blood vessels more permeable and promotes angiogenesis by increasing the levels of vascular endothelial growth factor (VEGF), which again is permissive for metastasis (73).

A deeper understanding of the molecular pathways linking inflammation and cancer could lead to the development or repurposing of targeted therapies that selectively interrupt tumor-driven inflammatory circuits without impairing antitumor immune surveillance (72). Inflammasomes, as critical components of the inflammatory response and focal coordinators of inflammation, are also associated with cancer initiation and progression (74).

### 4.2 Immune dysregulation and cancer progression

**Figure 3:**
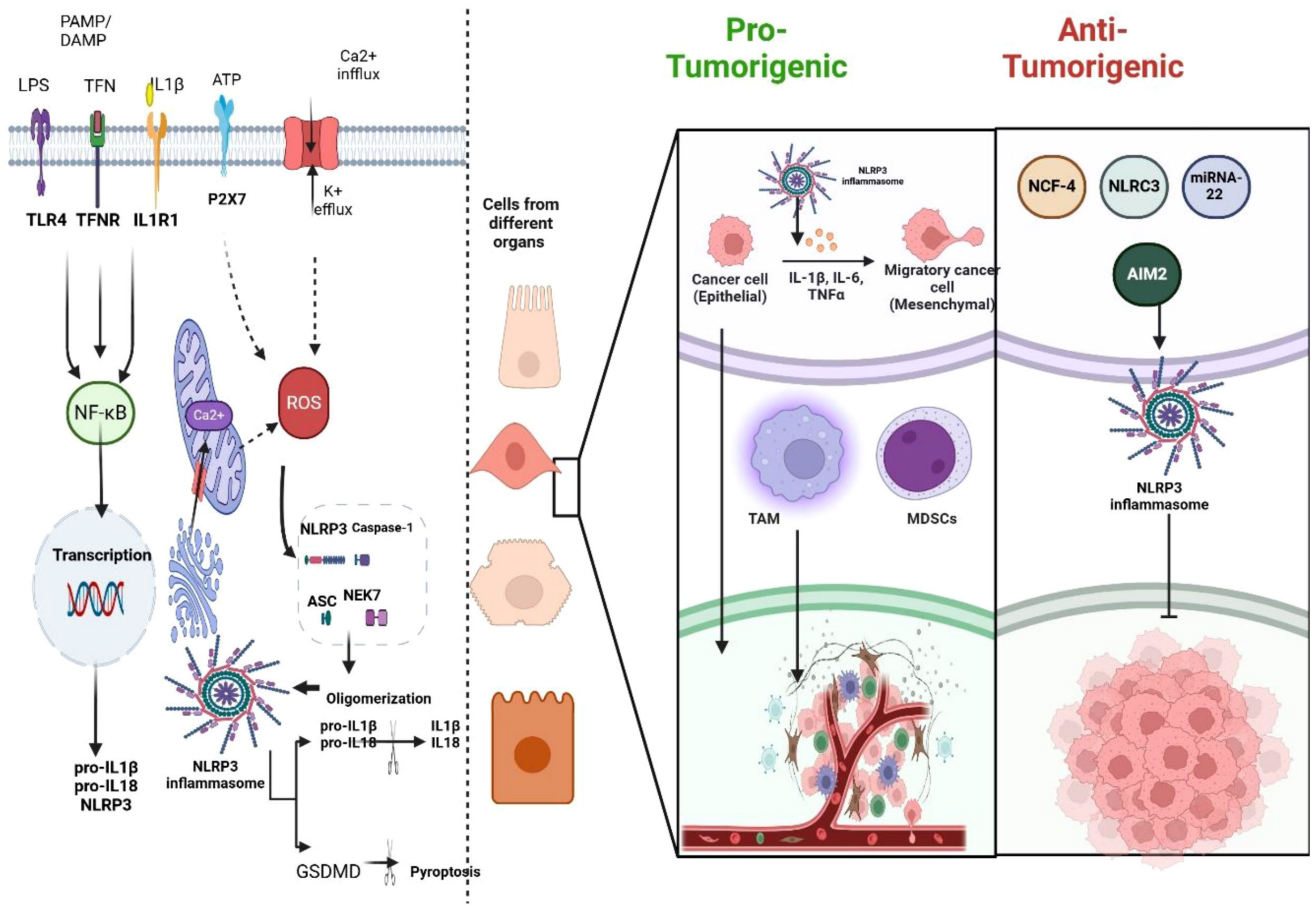
The mechanisms of action of the inflammasome in cancer and its dual role as both a pro-tumorigenic and anti-tumorigenic pathway. Figure created using BioRender.

Cancer is a complex disease that is often driven by chronic inflammation, which impacts cell plasticity and promotes mutations in normal or transformed cells (75, 76). Infection, dysregulated innate immune responses, or environmental factors often drive inflammation-induced neoplastic transformation (76–78). Inflammasomes are key inflammatory mediators activated in cancer and implicated in tumor initiation (79, 80). Inflammasome-related pathways are upregulated in various cancer types and play a role in both tumor development and progression (80, 81). Conversely, they are also associated with tumor suppression in certain contexts (81). In this section, we explore the pro-tumorigenic effects of the inflammasome in different cancer types while briefly discussing their potential protective role. **Figure 3** provides an overview of the contradictory roles of inflammasomes in cancer.

Inflammasomes influence malignant transformations throughout all stages of tumorigenesis, exerting either protective or tumor-promoting effects depending on the cancer type (13). Activated inflammasomes (e.g. NLRP3, NLRC4, AIM2) assemble caspase-1, which cleaves pro–IL-1β and pro–IL-18 into active cytokines and cleaves gasdermin proteins to execute pyroptosis (82, 83). The chronic release of proinflammatory cytokines from pyroptosis constructs and preserves an inflammatory milieu that promotes tumor growth (84). For instance, NLRP3 inflammasome was found to facilitate the invasion of myeloid cells into the TME such as tumor-associated macrophages (TAMs) and myeloid-derived suppressor cells (MDSCs), thereby supporting tumor progression (85). On the other hand, inflammasomes can also elicit tumor-suppressive roles in certain cancers like colon cancer, partly through IL-18. IL-18 has been proposed to play a critical role in the repair of colonic epithelium post-injury, by maintaining barrier integrity and attenuating inflammation. In an *in-vivo* study conducted in azoxymethane/dextran sodium sulphate (AOM/DSS) CAC colorectal cancer model, recombinant IL-18 has been reported to suppress tumor progression, suggesting that IL-18 is crutial in maintaining intestinal homeostasis and and protecting against colitis (86).

#### 4.2.1 Inflammasomes in colorectal cancer

Inflammasomes are implicated in colorectal cancer (CRC) initiation and progression. Wang et al. demonstrated that NLRP3 is upregulated in CRCs and is linked to advanced (stage III and IV) cancer and lymph node invasion, together with a possible correlation between NLRP3 and p-S6K1 expression in CRC tissues (87). As a result, the authors suggested that targeting NLRP3 could be a promising therapeutic approach for CRC patients resistant to mTORC1-targeted therapy, as there was an association between NLRP3 and the mTOR-S6K1 pathway (87). Li et al. investigated the effect of NLRP3 inflammasomes on colorectal cancer proliferation and progression and found that their inhibition reduced EMT and improved recurrence-free and progression-free survival. This effect was mediated by the S6K1-GLI pathway, another possible therapeutic target (88). Additionally, in a prognostic study, NLRP3 expression was higher in CRC than in adjacent tissues, with NLRP3 levels negatively associating with prognosis (89). In the same study, the authors investigated NLRP3 in the HCT116 CRC xenograft model and found that inhibition of NLRP3 expression significantly decreased transcription of IL-1β and IL-18, impaired proliferation, and promoted apoptosis (89). This study highlighted NLRP3 as a prognostic biomarker and a potential therapeutic target in CRC (89).

There have been several reviews of the role of inflammasomes in gastrointestinal and colorectal cancer (90–92). Li et al. demonstrated that activation of the inflammasome, particularly the NLRP3 subtype, by 5-hydroxytryptamine promoted cancer progression in CRC cell lines, an azoxymethane/dextran sodium sulfate–induced colorectal cancer mouse model, and colorectal cancer tissues (93). Deng et al. indicated a potential role for NLRP3 activation in macrophages and in colon cancer migration and liver metastasis through crosstalk with CRC cells. In the study, the authors highlighted the pro-tumorigenic potential of NLRP3 in CRC and its role as a therapeutic target. Wang et al. further provided evidence for the tumor-promoting potential of inflammasomes, demonstrating the induction of a pro-inflammatory tumor microenvironment *in vivo* through NLRP3 inflammasome activation by *Porphyromonas gingivalis*, which promoted CRC tumorigenesis (94).

In some other studies and contexts, inflammasomes have been shown to protect against CRC. Domblides et al. analyzed expression of NLRP6 and IL-18 in 104 human CRC tissues and demonstrated that lower expression was associated with worse CRC outcomes (a 26% five-year survival rate) and reduced numbers of infiltrating lymphoid cells in human CRC tissue samples (95). Interestingly, Li et al. examined the spatiotemporal effect of NCF4 on the inflammasome in CRC and found that NCF4, with the help of NCF1 and NCF2, activates inflammasome assembly at the perinuclear region of the cell (96). This activation activates CD8^+^ T cells and NK cells, suppresses precancerous and transit-amplifying cells, and enhances the inflammasome–IL-18–IFN-γ axis at the early stage of CRC. All these effects were reversed in an *Ncf4*^-^/^-^ mouse model (96). Notably, a study on human CRCs revealed a decrease in NLRP1, NLRP3, NLRC3, NLRC4, and AIM2 in cancer as well as a potential role for NLRC3 and AIM2 as biomarkers for disease progression. *In vivo* data also support an anti-tumorigenic role for inflammasomes in CRC (90, 91).

The outcome of inflammasome activation and its pleiotropic role in CRC therefore appear to be controlled by various signaling molecules and stage of activation, and further work is necessary to better understand contextual cell fate in terms of inflammasome activation.

#### 4.2.2 Inflammasomes in oral squamous cell carcinoma

Inflammasomes are also implicated in OSCC initiation, growth, and progression. Wang et al. investigated NLRP3 expression in OSCC cell lines, xenograft models, and human tissues, finding significant upregulation of NLRP3 expression in both OSCC cell lines and tissues and supporting NLRP3 as a potential therapeutic target in OSCC (97). Yao et al. provided new evidence on the crucial role played by the inflammasome/NLRP3 in oral cancer initiation and progression (98), demonstrating that periodontal bacteria, such as *Porphyromonas gingivalis* and *Fusobacterium nucleatum*, can promote cancer initiation and progression through overexpression of NLRP3 and activation of ATR-CHK1, leading to chromosomal instability due to uncontrolled S-phase arrest. The same study showed that cytokines such as IL-6, TNF-α, IL-18, ASC, and caspase-1 were upregulated. However, NF-κB, NOD-like receptors (NLRs), NLRP3, and IL-1β were suppressed, indicating a complex regulatory relationship (98). Additionally, T lymphocytes and macrophages, including CD4^+^ T cells, CD8^+^ T cells, and CD206^+^ macrophages, were increased (98). This highlights a sophisticated interplay between tumor-promoting inflammation and impaired signaling pathways, which may influence cancer progression. Clarifying this contradiction is crucial for understanding NLRP3’s role in oral cancer dynamics. A recent study by Jain et al. examined NLRP3 expression in tissue and blood from patients with OSCC and potentially malignant oral disorder (PMOD). The study established a significant correlation between expression of these factors in PMOD, cellular transformation, OSCC progression, and dedifferentiation (99).

Corroborating this, Feng et al. detected upregulation of NLRP3 expression and downregulation of miRNA-22 levels in OSCC compared with adjacent normal tissues. miRNA-22 inhibited OSCC cell viability, migration, and dissemination, but NLRP3 suppressed miRNA-22 expression to promote these processes (100). Wu et al. verified that ASC, an adaptor protein essential for inflammasome assembly (15), overexpression was associated with significantly worse OSCC disease stage, progression, overall survival, and disease-free survival. They found that tumor tissues upregulated ASC and, through ASC-associated inflammasomes, upregulated IL-1β, CASP1, and NLRP3. This was the first study to establish a role for ASC in OSCC migration and aggression and as a poor prognostic factor (101). Another confirmatory study by Wu et al. demonstrated that ASC contributes to OSCC lymph node metastasis through the ASC-HIF-1α pathway, paving the way for a new therapeutic target to manage OSCC progression (102).

Interestingly, NLRP3 is also involved in resistance to the chemotherapy 5-FU via the ROS/NLRP3 inflammasome/IL-1β pathway in both *in vitro* and *in vivo* models (103). Knocking out NLRP3 or caspase-1 in mice increased the sensitivity of OSCC cells to 5-FU, so targeting inflammasomes may be a means to enhance 5-FU efficacy as an adjuvant therapy in OSCC (103). Studies in a mouse model of head and neck squamous cell carcinoma provided evidence that a decrease in NLRP3 decreases tumor-associated macrophages (TAMs) and myeloid-derived suppressor cells (MDSCs) (104) and consequent depletion of cytokine-associated cancer markers like IL-1β, IL-6, IL-10, and CCL2. Therefore, NLRP3 expression in macrophages may be a biomarker of cancer progression and a poor prognostic factor (104). From the therapy perspective, Casili et al. showed that the specific NLRP3 inflammasome antagonist BAY-117082 decreased tumor growth in an orthotopic model of OSCC through regulation of EMT and a reduction in MMP2 and MMP9, mitigating cancer metastasis (105).

Therefore, in OSCC, inflammasomes have been shown to be associated with tumor initiation, progression, and metastasis through various downstream molecular pathways. Inflammasomes also play a crucial role in the interplay with the cell cycle and the activation of immune cells, which contribute to immune evasion and the establishment of an immunosuppressive cancer microenvironment. Consequently, targeting this pathway offers a promising therapeutic strategy for OSCC and could serve as a prognostic biomarker.

#### 4.2.3 Liver cancer (hepatocellular carcinoma, HCC)

Chronic liver diseases, including hepatitis B and C infection, alcohol-related liver disease, and metabolic dysfunction-associated steatohepatitis (MASH), are associated with activation of the NLRP3 inflammasome, which contributes to persistent inflammation and accelerates progression to hepatocellular carcinoma (HCC). Inflammasome-related cytokines like IL-1β and IL-18 increase liver injury by promoting collagen deposition and scar formation (106). Furthermore, hepatocyte cell death through pyroptosis, apoptosis, or necroptosis increases inflammation and fibrosis by releasing DAMPs, which sustain inflammasome activation. In alcohol-related liver disease and MASH, lipid accumulation and oxidative stress trigger inflammasome activation, leading to hepatocyte pyroptosis (107).

Like in CRC, NLRP3 inflammasomes are pleiotropic in HCC. On the one hand, their activation leads to cancer cell pyroptosis and an anti-tumor immune response, thereby contributing to tumor suppression. On the other, when NLRP3 is chronically or improperly activated, it creates a microenvironment that facilities tumor development through sustained inflammation, increased ROS production, and activation of NF-κB signaling, all of which support cancer cell survival, growth, and immune evasion (108).

Furthermore, NLRP3 is closely associated with several important oncogenic signaling pathways including NF-κB, MAPK/ERK, and AMPK/mTOR. Dysregulation of these signaling cascades through NLRP3 activation may increase inflammatory signals and metabolic stress, leading to hepatocarcinogenesis (109, 110). In addition, there is crosstalk between ERβ/MAPK/ERK signaling and NLRP3, which influences immune activity and the development of fibrosis in the tumor microenvironment. Together, these interactions highlight NLRP3 as a central node in the complex network that links inflammation, metabolism, and liver cancer progression (107, 108, 111). Therapeutic strategies that modulate NLRP3 activation may therefore offer the opportunity to control HCC progression while preserving advantageous anti-tumor immunity.

#### 4.2.4 Lung cancer

Lung inflammation, arising from various environmental factors like tobacco smoking, air pollution, or persistent lung infections, is a key contributor to lung cancer development. The inflammatory microenvironment promotes carcinogenesis through oxidative stress, cytokine-mediated signaling, and immune modulation, facilitating tumor initiation, progression, and metastasis (112, 113).

##### 4.2.4.1 Air pollution and lung cancer

Air pollution exposure to particulate matter (PM2.5: 2.5 microns), microplastics, and industrial pollutants is strongly associated with lung inflammation and an increased risk of lung cancer. Air pollutants cause lung epithelial cell damage, oxidative stress, and disturbances in normal cellular function that create a tumor-promoting microenvironment. Long-term inhalation of air toxins also induces chronic activation of NLRP3 inflammasomes, developing sustained inflammatory reactions that promote carcinogenesis (113).

##### 4.2.4.2 The role of NLRP3 inflammasomes in lung cancer

Lung cancer remains the most prevalent cause of cancer mortality worldwide, and non-small cell lung cancer (NSCLC) represents approximately 85% of all cases. The NLRP3 inflammasome contributes to inflammation-mediated tumorigenesis, immune regulation, and cell death mechanisms like pyroptosis (114, 115).

Induction of pyroptosis in NSCLC can effectively suppress tumor growth even in cancer cells resistant to apoptosis. Polyphyllin VI (PPVI), a bioactive compound isolated from *Trillium tschonoskii* Maxim (TTM), induced pyroptosis in NSCLC via the ROS/NF-κB/NLRP3/GSDMD pathway, indicating the potential of pyroptosis-inducing therapy for the treatment of lung cancer (116).

##### 4.2.4.3 NLRP3 activation and lung epithelial cell transformation

Activation of NLRP3 inflammasomes has been implicated in the transformation of lung epithelial cells through IL-1β and IL-18 signaling, which induces a pro-tumor inflammatory microenvironment. Chronic treatment with inflammatory stimuli induces long-term NLRP3 activation, inducing EMT by modulating Snail and Twist, thus facilitating tumor growth and immune evasion. IL-1β plays a crucial role in promoting tumor angiogenesis and proliferation through activation of NF-κB and MAPK signaling. Preclinical models have shown that blockade of IL-1β signaling with monoclonal antibodies can reduce tumor burden and metastasis, further illustrating the role of NLRP3-mediated inflammation in lung cancer development. Finally, the NLRP3 inflammasome again plays a dual role in the development of lung cancer, as its activation not only facilitates inflammation-mediated tumor growth but targeted modulation of NLRP3 signaling can reverse immune evasion by tumor cells (115, 117, 118).

#### 4.2.5 Breast cancer

##### 4.2.5.1 Inflammasomes and IL-1β activation in breast cancer progression

Inflammasomes, particularly NLRP3, play a crucial role in breast cancer progression, influencing inflammation, immune evasion, metastasis, and therapy resistance. IL-1β downstream of inflammasome activation contributes to tumor growth, EMT, and immune suppression, while IL-18 is associated with a poorer prognosis and enhanced metastasis (119).

Since inflammasomes upregulate immune checkpoint molecules like PD-L1, they contribute to immune evasion and therapy resistance. While no direct anti-inflammasome therapies currently exist, targeting NLRP3, IL-1β, or purinergic signaling has shown potential for reducing tumor progression and overcoming treatment resistance. High levels of ATP released from radiotherapy-resistant breast cancer cells have been shown to stimulate inflammasome activation through purinergic receptors like P2Y2R. This activation leads to increased secretion of IL-1β and IL-18, which promote angiogenesis, tumor cell migration, and resistance to chemotherapy and radiotherapy. Additionally, inflammasomes have been linked to increased expression of the proliferation marker Ki-67, suggesting a role in enhancing tumor aggressiveness (119, 120).

Yan et al. recently demonstrated that cisplatin (DDP) induces pyroptosis in triple-negative breast cancer (TNBC) cells via activation of the MEG3/NLRP3/caspase-1/GSDMD signaling pathway. In this mechanism, cisplatin upregulates expression of the lncRNA *MEG3*, which subsequently activates the NLRP3 inflammasome and consequent cell death (121).

## 5 Inflammasome activation: a common pathway in ARDS and cancer

### 5.1 Systemic inflammation in ARDS and cancer: a shared pathway

**Figure 4:**
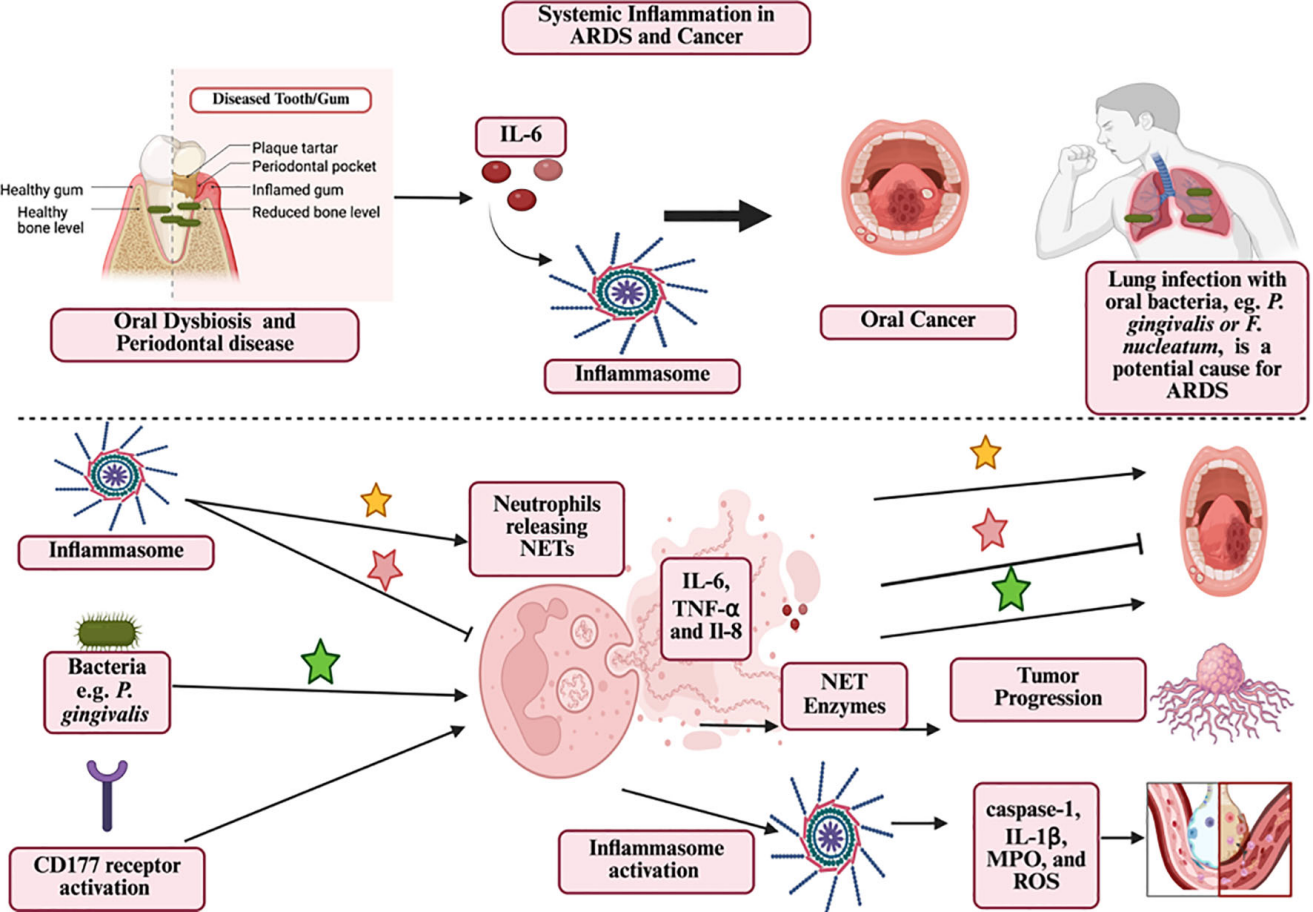
The mechanisms by which systemic inflammation can contribute to the development of ARDS and cancer. Oral dysbiosis and periodontal disease can initiate systemic inflammation, characterized by the release of IL-6 and the activation of inflammasomes. Inflammasome activation can either induce or inhibit NET formation, consequently promoting or inhibiting cancer progression. Pathogenic bacteria such as *P. gingivalis* and *F. nucleatum* can induce neutrophil extracellular traps (NETs), leading to the release of pro-inflammatory cytokines (IL-6, TNF-α, IL-8). These pathways promote tumor progression, metastasis, and the initiation of ARDS. Additionally, inflammasome activation through CD177 receptors amplifies the inflammatory response by inducing caspase-1, IL-1β, MPO, and ROS production, further contributing to cancer invasion and ARDS progression. Figure created using BioRender.

The preceding discussion highlights that inflammasomes are common to the pathogenesis of both cancer and lung diseases, including ARDS. We propose that the shared pathways and mechanisms in cancer and ARDS could represent a useful paradigm for preventing lung diseases/ARDS caused by cancer and explaining how lung diseases/ARDS create a permissive environment for cancer initiation and progression. **Figure 4** illustrates the causes and mechanisms by which systemic inflammation contributes to the development of ARDS and cancer.

#### 5.1.1 Bacteria as a cause of cancer and ARDS

There is clear evidence of the existence of an oral-lung axis and that oral health impacts lung function (122–124). *Porphyromonas gingivalis*, a pathogenic bacterium that colonizes periodontal pockets (125), has is associated with the initiation and progression of OSCC (126), and contributes to chemotherapy resistance (127, 128). In human respiratory epithelial cells, *P. gingivalis* enhances the production of IL-6 and IL-8, surpassing even levels induced by *S. pneumonia* (129, 130). This increase in proinflammatory cytokine production appears to be governed by TLR-2 receptors (129). IL-6, a key driver of cytokine release syndrome and ARDS, has been extensively studied in the context of COVID-19-induced ARDS (131, 132). Notably, IL-6 initiates and promotes the proliferation and dissemination of lung (133) and oral cancer (134) cells via the NLRP3 inflammasome pathway. Oral bacteria related to periodontal disease and oral cancer can invade the lung, increasing the risk of ARDS through inflammasomes. Consequently, treating periodontal disease and making the oral environment unsuitable for bacteria to thrive and persist could help to prevent oral cancer development and the lung infections that lead to ARDS through inflammasome activation. Notably, therapies that target the disrupted cytokine signature may help ameliorate ARDS, prevent cancer formation, or enhance its regression.

#### 5.1.2 Neutrophil extracellular traps as a cause of cancer and ARDS

Neutrophils and NET formation play a crucial role in both ARDS and cancer; however, inflammasome activation extends beyond neutrophils. It also includes a variety of cell types, such as macrophages, epithelial cells, endothelial cells, and other immune cells, depending on the disease (4, 135, 136). It’s important to note that the connection between NETs and inflammasome signaling is not straightforward (137–140). NET formation can transpire both upstream and downstream of inflammasome activation and may represent a concurrent inflammatory pathway in certain contexts, initiated by prevalent triggers such as infection, ROS, and DAMP (140).

NETs are characterized by a scaffold of decondensed chromatin and other proteins released extracellularly by neutrophils as a defense mechanism against pathogens (141). This process is considered a double-edged sword, as NETs not only play a role in immune protection and pathogen clearance but also contribute to the underlying etiology of certain inflammatory diseases and cancer progression and dissemination (141).

The interplay between NETs and NLRP3 inflammasome is considered a key inflammatory axis contributing to multiple inflammatory conditions (142, 143). Mechanistically, the activation of the NLRP3 inflammasome and NET formation (NETosis) are interconnected. Neutrophils start releasing NETs in certain conditions when phagocytosis fails to contain inflammation (144, 145). These NETs can amplify inflammasome signaling in macrophages through activating caspase-1, the key effector enzyme of the inflammasome in macrophages, leading to the production of IL-18 and IL-1β. Conversely, NLRP3 can also induce NET formation through producing active IL-1β and IL-18 pro-inflammatory cytokines. IL-18 in particular is known to induce NETosis in human neutrophils. Together, these observations suggest increased inflammasome activation secondary to the enhanced NETs formation (146).

Saito et al. demonstrated NET inhibition increases tumor-infiltrating lymphocytes in CRC (147). Kim et al. reported that a multi-herbal decoction mitigated ALI by blocking the MAPK, NF-κB, and NLRP3 pathways and the production of cytokines that prime and promote neutrophils to release NETs (148). *P. gingivalis* can also promote cancer progression and metastasis by enhancing NET production in the TME, thereby facilitating cancer cell survival (149). Guo et al. demonstrated that the presence of *P. gingivalis* correlates with NET markers, promoting cancer progression and metastasis. This study suggested that targeting NETs may help ameliorate OSCC (149). Targeting *P. gingivalis* through the treatment and prevention of periodontal disease may help to prevent cancer and reduce the risk of ARDS.

In the context of inflammasomes, particularly NLRP3, a recent study by Zhai et al. revealed a critical role for NLRP3-mediated pyroptosis activation in inhibiting NET induction and OSCC metastasis (150). They demonstrated that high levels of NETs serve as a poor prognostic marker in OSCC, suggesting that NETs may promote cancer progression through NET-related enzymes like neutrophil elastase (150). However, NLRP3 protected against this process (150). Notably, they observed NLRP3 overexpression in primary tumors and NLRP3 downregulation in metastatic ones, positioning NLRP3 and NETs as potential prognostic markers for cancer dissemination and offering new insights into therapeutic strategies for OSCC. In OSCC patients, particularly those with stage III/IV disease, neutrophils are more prone to produce NETs. This NET formation is associated with elevated levels of proinflammatory cytokines such as IL-6, IL-8, and TNF-α (151). Li et al. demonstrated that NETs induce a procoagulant phenotype in endothelial cells, which correlated with the heightened hypercoagulable state observed in these patients (151). Future studies should focus on elucidating the mechanisms by which NETs activate the coagulation pathway to develop targeted strategies to inhibit this process and potentially impede cancer progression and metastasis. In both orthotopic 4T1 breast cancer and invasive HCT116 colon cancer metastatic mouse models, targeting NETs using cationic poly (aspartic acid)-based nanoparticles significantly reduced liver and lung metastases (152). These results underscore NETs as a promising therapeutic target in advanced solid tumors, with the potential to reduce metastasis and associated complications such as ARDS and disease progression-related health deterioration.

In hyperinflammatory ARDS, neutrophils secreting NETs are key drivers of severe and fatal disease (153). In both *in vitro* and *in vivo* mouse models, CD177 was identified as a novel neutrophil target, and blocking CD177 significantly reduced the production of inflammatory cytokines like caspase-1, IL-1β, MPO, and ROS, primarily by inhibiting NLRP3 inflammasome activation (153). These results raise the prospect of targeting CD177 to mitigate ARDS progression and suggest a new strategy for disease management by limiting neutrophil-driven inflammation. In this context, Qu et al. confirmed overexpression of NETs in sepsis-induced lung injury/ARDS and linked this to impaired autophagic flux, a process essential for maintaining normal physiological responses (154). Their study proposed that inhibition of autophagic flux occurs through upregulation of METTL3-mediated m6A RNA modifications, which leads to the degradation of SIRT1, a key regulator of autophagy (154). These findings add to a growing body of evidence highlighting a critical role for NETs in ARDS pathogenesis and support the therapeutic potential of targeting NET formation and its upstream regulators. Consistent with this, another mechanistic study further elucidated the role of NETs in promoting ARDS progression (155). Li et al. used a combination of pharmacological inhibitors to suppress NET formation and assessed ARDS severity in bronchoalveolar lavage fluid (BALF) from lipopolysaccharide-exposed mice (155). Treatment with DNase I (a NET DNA-degrading enzyme), BB-Cl-amidine (a NET formation inhibitor), and Ac-YVAD-cmk (a pyroptosis inhibitor) reduced alveolar macrophage pyroptosis and decreased neutrophil–macrophage interactions within alveoli (155). These interventions also diminished pro-inflammatory cytokine levels, reduced AIM2 expression, and alleviated ARDS symptoms (155). These findings suggest that NETs aggravate disease progression by activating inflammasomes, which drive cytokine release and pathological interactions among innate immune cells.

Therefore, targeting NETs in ARDS and cancer may be an effective approach to slow or halt disease progression. In cancer, NETs activate pyroptosis via inflammasome activation, thereby initiating tumor progression and metastasis (156). Furthermore, there is increasing evidence of an interaction between NETs and inflammasomes in ARDS (157) and cancer (158). For instance, Wang et al. showed that NETs promote NSCLC metastasis by increasing EMT in a way that depends on NF-κB and NLRP3 (139). Mechanistically, NET activation can occur through gasdermin (159), a downstream effector protein of the inflammasome that mediates pyroptosis and IL-1β release (160, 161). In addition, several therapies, many of which target the inflammasome, inhibit NETosis and suppress cancer progression (162).

Taken together, these findings indicate that NETs are an important component of the inflammatory network shared by ARDS and cancer, but their contribution should be interpreted within a broader cellular context rather than as a neutrophil-exclusive mechanism. Notably, the functional consequences of NETs differ between ARDS, where they contribute primarily to acute tissue injury, and cancer, where they promote metastasis, immune modulation, and thrombosis. This further emphasizes that the contribution of NLRP3 and associated inflammatory pathways is highly context dependent across these two disease settings.

### 5.2 Comparative analysis: shared and distinct mechanisms

#### 5.2.1 Similarities in inflammasome activation pathways

NLRP3 is the most extensively studied and best-characterized inflammasome in both ARDS and cancer (74). It is activated by a broad range of stimuli relevant to both disease contexts, including ROS, DAMPs, mitochondrial dysfunction, and infection- or injury-related cellular stress (4, 163). Because these triggers are shared across acute lung injury and tumor-associated inflammation, NLRP3 was selected in this review as the principal mechanistic focus at the ARDS-cancer interface. Nevertheless, other inflammasomes, including AIM2 and NLRC4, have also been implicated in both lung injury and tumor biology (4, 163, 164). Accordingly, NLRP3 should be viewed as a central, but not exclusive, component of a broader inflammasome network involved in these conditions.

Based on the premise that there is a potential association between ARDS and cancer (165–167), in this section we explore the details of their relationship through shared signaling pathways specific to inflammasomes. Based on our assessment of the available literature, the relationship between both diseases appears to be bidirectional, i.e., cancer can contribute to ARDS onset and, conversely, through inflammasomes, ARDS can initiate or exacerbate cancer progression through inflammasome pathways. For instance, it is well documented that inflammation contributes to mutagenesis through various mechanisms involving RONS, resulting in DNA damage and cancer development (70). A substantial amount of experimental evidence from animal models indicates the importance of RONS in the pathogenesis of ARDS (168). In the context of inflammasome pathways, several inflammasomes, mainly NLRP3, are implicated in this transition. Conversely, NLRP3 is also implicated in ARDS. The severe clinical complications seen in ARDS due to lung injury and inflammatory responses can at least in part be explained by NLRP3 overactivation. NLRP3 releases proinflammatory cytokines in response to injury, infection, or stress that amplify tissue damage and perpetuate injury and inflammation (169–172). Furthermore, increased levels of NLRP3 in ARDS patients are associated with lower PaO_2_/FiO_2_ ratios, reflecting greater disease severity (170). While direct evidence linking cancer and ARDS development through NLRP3 is limited, pharmacological and radiation therapies used in cancer and tumor-derived factors could potentially establish such a relationship (173).

Various anticancer therapeutics induce ALI (174). For instance, Sherif et al. demonstrated that the anticancer drug methotrexate significantly upregulates lung mRNA and protein expression of NLRP3, ASC, and caspase-1 (175). Another study by Han and co-workers confirmed radiotherapy-induced ALI and expression of NLRP3 in mouse models (176). Interestingly, Rao et al. also evaluated radiation-induced lung injury (RILI) and found that NLRP3 was the highest-ranking inflammasome in the process. Its activation led to the release of IL-1β, which was observed to promote fibroblast activation, migration, and proliferation and, therefore, accelerated RILI (115).

Tumor-derived factors are also important to consider in relation to cancer progression and ARDS. Molecules secreted from tumors can reach distant organs such as the lung either though metastasis or the circulation. For example, dying tumor cells release adenosine triphosphate (ATP), which can trigger NLRP3 inflammasome activation by acting on P2X7 purinergic receptors (P2RX7) (177). Furthermore, ROS produced by cancer cells can also activate inflammasomes through ERK1/2 and MAPK signaling (178, 179). Other factors released from dying tumor cells and implicated in inflammasome inflammatory activities include exosomes and high-mobility group box 1 (HMGB1) (180, 181). Therefore, understanding how cancer-derived factors modulate lung inflammation via the inflammasome axis may reveal new inter-organ signaling pathways.

**Figure 5:**
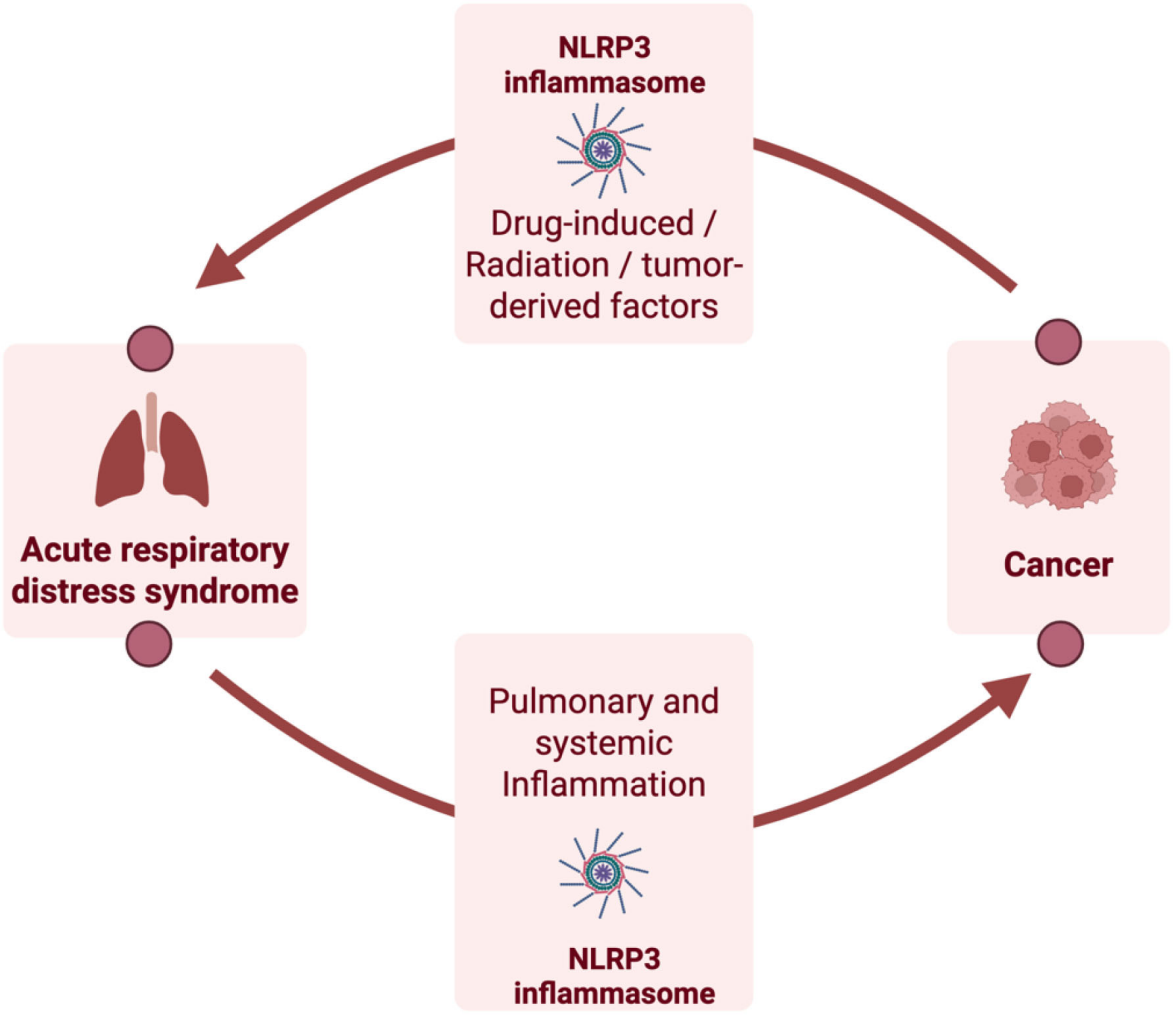
Schematic showing the bidirectional relationship between ARDS and cancer through NLRP3 inflammasomes. Figure created using BioRender.

In contrast, chronic inflammation propagated by lung diseases such as ARDS can promote tumor cell proliferation and invasion. In a recent study, Pooladanda and co-workers investigated the mechanism by which acute lung inflammation can activate the TME, concluding that the released inflammatory cytokines created favorable conditions for tumor development in the lungs (182). Interestingly, NLRP3 inflammasomes have been shown to be pro-tumorigenic in numerous cancers such as lymphoma, as evidenced by the *in vivo* NLRP3 blockade that resulted in the inhibition of lymphoma growth (136, 183). **Figure 5** summarizes the bidirectional relationship between ARDS and cancer.

Future studies, including longitudinal clinical investigations and mechanistic experimental models, will be required to determine whether acute lung injury and associated inflammasome activation can meaningfully influence tumor initiation, progression, or metastasis.

Clinically, ARDS is recognized as a complication in patients with cancer and this is highlighted in both narrative and prospective clinical studies such as the review by Young and Shannon (184) and the YELENNA prospective multinational observational cohort study (185). These reports support an important point of intersection between acute lung injury and malignancy care (184, 185). Nevertheless, this relationship appears to be driven mainly by treatment- and disease-related factors rather than by a direct mechanistic continuum between the two conditions.

Crucially, the proposed intersection between ARDS and cancer should be interpreted with caution. This is particularly important given the distinct temporal dynamics of these conditions, as ARDS represents an acute inflammatory syndrome (186), whereas carcinogenesis is a chronic, multi-step process that develops over years (187). As such, the current evidence does not support a direct temporal progression from ARDS to cancer. However, it is important to note that, despite its acute onset, ARDS can predispose patients to long-term chronic lung damage. This is evidenced by the reported persistence of respiratory symptoms for up to 12 months post-ARDS, significant impairments in pulmonary function, which may be severe in some patients, along with interstitial damage and peristent lung fibrosis (188, 189). Lung fibrosis can promote tumor progression through dysregulations in TGF-β, a central signalling pathway that links fiberosis to the progression of cancer and facilitaes immune tolerance and tissue repair (182, 190). at immune and microenvironmental levels, the persistent activation of TGF-β signaling drives ECM remodeling and fibroblast activation, as well as induces strong immunoregulatory effects that together create an immunosuppressive environment conducive to tumor initiation and progression (190). Nevertheless, we acknowledge that the long-term consequences of ARDS and their relationship to cancer are still interesting areas of research and requiring further exploration. Pooladanda et al. examined the mechanism by which acute lung inflammation can enhance lung metastasis and activate tumor microenvironment in *in-vivo* and *in-vitro* LPS-induced models. They reported that the cytokine storm influenced oxidative stress as well as nitrosative stress through upregulation of cellular nitrite levels in mice macrophage. This created a permissive environment supportive of the establishment of tumor microenvironment in the lungs. In addition, LPS-induced lung inflammation was associated increases in the expression of the epigenetic regulator bromodomain 4 (BRD4), which in turn upregules p65 and STAT3 signalling molecules, thereby contributing to the inflammation-driven tumor metastasis (182). Although accumulating evidence indicates that inflammasome activation, particularly involving NLRP3, contributes to inflammatory signaling in both ARDS and tumor biology, direct mechanistic evidence supporting ARDS as a causal driver of malignancy remains limited (4, 79, 135, 191). Much of the currently available literature reflects shared inflammatory pathways, such as reactive oxygen species production, cytokine release, and immune cell recruitment, rather than longitudinal or mechanistic studies demonstrating a direct progression from ARDS to cancer (21, 185, 192, 193). Collectively, the proposed ARDS-cancer inflammasome axis should therefore be viewed as a hypothesis-generating conceptual framework that integrates converging evidence from pulmonary inflammation and tumor immunology. Future studies, including longitudinal clinical investigations and mechanistic experimental models, are needed to determine whether ARDS or acute lung injury can meaningfully contribute to tumor initiation, progression, or metastasis.

## 6 Hypothetical framework for an ARDS-cancer-inflammasome axis

### 6.1 Cancer therapies as a driving force for ARDS/ALI

**Figure 6:**
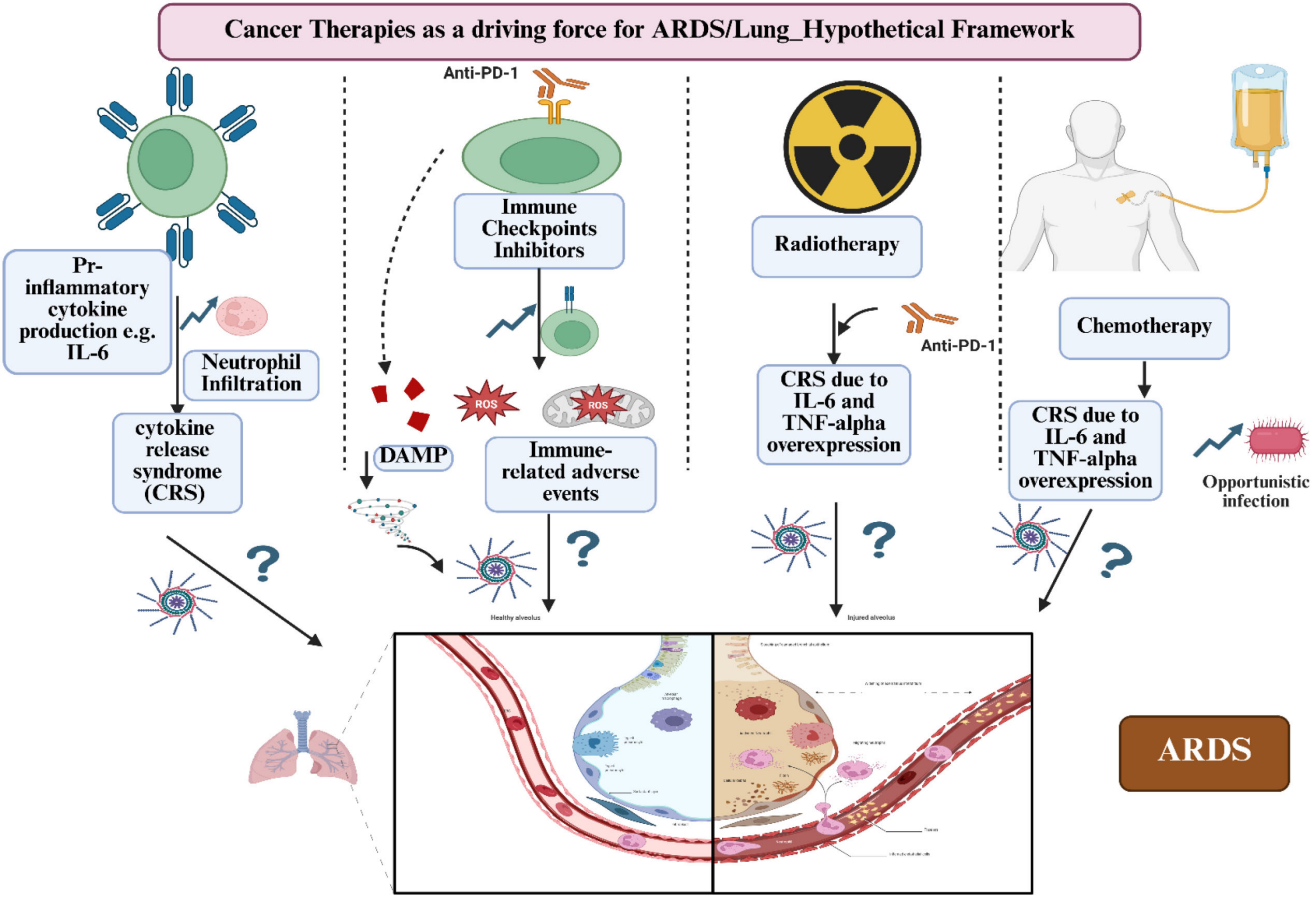
A hypothetical framework for how cancer therapies can induce ARDS. Figure created using BioRender.

Conventional therapies and newer immunotherapeutics often cause adverse events, among which ARDS is particularly significant (184, 194). ARDS can also arise in cancer patients due to opportunistic bacterial or fungal infections disseminating to the lungs on a background of immunosuppression, further compromising clinical outcomes (195, 196). In this section, we explore the molecular mechanisms by which cancer and its treatments can lead to ARDS. We pay particular attention to the genes disrupted by cancer therapies and that may serve as potential targets for preventing or mitigating ARDS in this vulnerable population. **Figure 6** illustrates a hypothetical framework depicting the mechanisms by which cancer therapies may induce ARDS.

CAR-T cells, an immunotherapeutic modality approved for hematologic malignancies (197) and currently being evaluated for solid tumors (198), are associated with several severe adverse events (199). Among these, cytokine release syndrome (CRS) is particularly notable (199, 200). CRS is characterized by a surge in pro-inflammatory cytokines that promote neutrophil infiltration, which can lead to increased alveolar inflammation, excessive immune cell recruitment, and ultimately, the development of ARDS. To mitigate or prevent this complication, IL-6 antagonists such as tocilizumab and corticosteroids may be administered to select cancer patients (201–203). Additionally, CAR-T cells targeting PD-L1 and mesothelin (MSLN) have been associated with lung toxicity, including lung injury, hypoxia, and pleural effusions (204, 205). While there is currently no explicit evidence that CAR-T cells induce ARDS through inflammasome pathways, we speculate that toxicity caused by this treatment modality may contribute to ARDS, potentially via NLRP3 activation. Taken together, further studies are needed to explore this potential link and to evaluate targeted therapies (e.g., IL-1β blockade) for prevention.

Immune checkpoint inhibitors (ICIs) are a breakthrough immunotherapeutic modality that have revolutionized cancer therapeutics and shown promising results in defined subsets of cancer, such as MSI/d-MMR CRC (198, 206–208). ICIs target inhibitory signals that lead to T cell suppression and tumor evasion from immune surveillance (206, 209). Anti-PD-1/PD-L1, but not anti-CTLA-4 ICIs, are most commonly implicated in lung toxicity (210–212). Although ICIs have improved overall survival and prolonged progression-free survival in specific groups of patients (213–215), immune-related adverse events (irAEs) such as pneumonitis, acute interstitial pneumonia, and ARDS are a known complication. Mechanistically, ICIs can be considered to activate T cells (216), which in turn may excessively produce ROS and induce mitochondrial dysfunction (217–219). This can consequently lead to inflammasome activation and the development of ARDS (220, 221). Consistent with this, ICIs can also induce cytokine storms through the production of DAMPs, which could result in inflammasome activation in lung macrophages and epithelial cells, thereby promoting ARDS (222). Targeting this pathway, particularly using inhibitors of cytokines downstream of inflammasomes, could be beneficial in preventing cancer treatment-related adverse events. However, as discussed above, targeting inflammasome pathways requires careful resolution of whether the effect might be pro- or anti-tumorigenic or contribute to treatment resistance, given the demonstrated pleiotropic effects in individual tumor types.

Radiotherapy is a conventional cancer treatment that destroys proliferating cells by damaging their DNA, thereby inhibiting cell division and preventing further tumor growth (223, 224). This modality is widely used in oncology and has proven efficacy for many cancer types. However, radiotherapy has its own drawbacks, especially lung injury, and it is a known contributor to the development of ARDS (225). Studies have shown a paradoxical effect of radiotherapy on the TME, turning immunosuppressive tumors into immunoactive ones, and vice versa (226). Modulating immune activity through radiotherapy, particularly activating it within the TME, plays a crucial role in enabling the immune system to destroy the tumor. One such immunological response includes the production of IL-1β through NLRP pathway activation (226). Nevertheless, radiotherapy has also been reported to cause adverse immune effects through systemic immune activation, which can manifest as CRS, ultimately worsening the patient’s condition and increasing mortality risk (226–228). Barker et al. reported a case involving a 65-year-old patient with untreated chronic lymphocytic leukemia given anti-PD-1 immunotherapy who developed CRS following radiotherapy, as evidenced by a tenfold increase in IL-6 and TNF-α levels every time tested one hour after treatment (227). Another study reported grade 4 CRS in a patient with metastatic TNBC previously treated with immunotherapy who presented with markedly elevated IL-6 (228). In these scenarios, elevated IL-6 levels may reflect broader inflammatory activation that can coexist with NLRP3 inflammasome signaling and IL-1β production. It is important to note that IL-6 is a pleiotropic cytokine that can be induced by a wide range of inflammatory mediators and signaling pathways, including NF-κB, and therefore should not be interpreted as a specific marker of NLRP3 inflammasome activation.

Immune-related complications in the form of CRS may contribute to the development of ARDS. Further work is now required to establish the underlying mechanisms.

## 7 Clinical implications and therapeutic opportunities

### 7.1 Targeting inflammasomes in ARDS

Understanding the link between ALI and NLRP3 inflammasomes could help in the development of vital new approaches for the treatment of ALI (135). One recent treatment strategy has been to inhibit the NF-κB pathway using drugs like propofol, which impacts pulmonary injury and damage. In the setting of lipopolysaccharide (LPS)-induced ALI, sevoflurane and propofol exposure was anti-inflammatory. In newborn rats, propofol effectively counteracted the impacts of LPS on the inflammasome and p38 MAPK/NF-κB pathways. This suggests that propofol could be useful for the treatment of ventilator-induced ALI in newborns (229, 230). Metformin has also been shown to provide protective benefits for the lungs in various ALI scenarios, as it reduces LPS-induced ALI by preventing endothelial cell pyroptosis by blocking NF-κB-NLRP3 signaling (231). LPS-induced inflammatory lung damage compromises the barrier function of the pulmonary endothelium (232). The anti-inflammatory agent berberine helps to modulate NF-κB signaling to suppress inflammation and NLRP3 inflammasome activation by the influenza virus in macrophages by improving mitochondrial autophagy and reducing mitochondrial ROS, thus mitigating lung damage (233). Glycyrrhizin is known for its antiviral and anti-inflammatory properties, reducing the production of IL-1β and TNF-α, inhibiting NF-κB activation, and modulating autophagy through the PI3K/AKT/mTOR pathway to improve LPS-triggered ALI (234, 235). It also inhibits NF-κB/NLRP3 inflammasome signaling, hence reducing the inflammatory reaction induced by LPS (236).

Another therapeutic approach is to inhibit the excessive ROS production that drives ALI. The anti-inflammatory emodin has been shown to improve LPS-induced ALI and suppress ROS, myeloperoxidase (MPO), and malondialdehyde (MDA) generation (237). In addition, emodin protects against LPS-induced ALI, presumably by reducing ROS generation and suppressing NLRP3 expression (237). Nrf2 plays a dual role as a transcription factor in the antioxidant response, inhibiting both inflammation and oxidative stress. Recent studies indicate that Nrf2 can prevent activation of the NLRP3 inflammasome, thereby alleviating LPS-triggered ALI. Oridonin (Ori) is an antioxidant and anti-inflammatory agent that activates Nrf2 (238). Ori can reduce LPS-induced inflammation via the NF-κB pathway and prevent activation of NLRP3 inflammasomes by disrupting the NLRP3-NEK7 axis (238).

Inhibiting mitochondrial autophagy is also a valuable treatment option for ALI. Mitochondrial-selective autophagy plays a vital role in eliminating damaged mitochondria to maintain mitochondrial homeostasis, preventing hyperinflammation induced by NLRP3 inflammasome activation (239). Sestrin2 (Sesn2) plays a significant role in cellular stress reactions and antioxidant protection. Sesn2 has been shown to prevent sepsis by initiating mitochondrial autophagy and inhibiting NLRP3 activation in macrophages (240). Sesn2 protects the lungs from LPS-induced ALI by preserving mitochondrial homeostasis during mitochondrial autophagy through Pink1/Parkin signaling. Treating ALI by inhibiting ion channels is an effective therapeutic approach. Calcium, a crucial intracellular secondary messenger, plays a vital role in several cellular mechanisms, and calcium influx can activate NLRP3 inflammasomes. CaM kinase (CaMK) is activated through binding of calcium to calmodulin (CaM), which induces inflammation. CaMK4 initiates the NLRP3 inflammasome, exacerbating lung damage, so KN-93, which inhibits CaMK4, improves ALI by inhibiting NLRP3 inflammasome activation (241). Rapamycin is a natural immunosuppressive agent that suppresses autophagy by inhibiting mTOR and thus the production of IL-1β and IL-18. Autophagy induction limits the secretion of IL-1β and IL-18 by eliminating damaged mitochondria and preventing the release of mitochondrial ROS (241). Further, rapamycin protects mice from LPS-induced lung damage by inhibiting mTOR activity, which subsequently decreases IL-1β and IL-18 production, inhibiting immune cell infiltration.

### 7.2 Inflammasome modulation in cancer therapy

Recent studies have shed light on the role of inflammasomes in cancer and the potential for inflammasome-targeting drugs. There have been major advances in cancer immunotherapy over recent years, exploiting various modes of action. Inflammasomes play an important role in the regulation of tumor immunity, as the inflammatory response can frequently stimulate the maturation of professional antigen-presenting cells (APCs) such as dendritic cells (DCs). Hence, inflammasomes can be targeted or modulated to achieve tumor immunotherapy.

Several therapeutic approaches that target inflammasome activity are now available, but poor specificity and low efficiency limit the use of synthetic compounds or small molecule inhibitors for this purpose. The most successful therapeutic approach has been to inhibit cytokine signaling (74). Glyburide (glibenclamide) is a sulfonylurea used to treat type 2 diabetes mellitus (242) that has also been used off-label for gestational diabetes in some settings (243). Beyond glycemic control, glyburide has been investigated as an anti-inflammasome agent (244), as it is reported to inhibit NLRP3 activation via upstream ion-flux modulation (244). Glyburide is therefore being explored for repurposing in acute lung injury/ARDS (245) and in select cancer models (246). These non-glycemic applications remain investigational and are not standard of care. Other treatment modalities targeting inflammasomes include MCC950, which prevents both typical and atypical NLRP3 inflammasome activation and the secretion of IL-1β in a mouse model of experimental autoimmune encephalitis (EAE). However, MCC950 helps to both alleviate EAE symptoms and inhibit tumor development, indicating that it can be used for treating NLRP3 inflammasome-related cancer.

Finally, there have been developments in novel nanomaterials for drug delivery, and nano-drug delivery systems are now widely used in immunotherapy and oncology as they help to improve stability and reduce toxicity (247). However, the physicochemical properties of nanoparticles can lead to inflammasome activation. For example, amine-modified polystyrene nanoparticles can both trigger NLRP3 inflammasomes and decrease the release of pro-inflammatory IL-1β from human macrophages (248). An anti-tumor vaccine based on a proton-driven deformable nano-delivery system has been proposed to efficiently deliver antigen peptides into the cytoplasm of immune cells and enhance innate immunity by acting as an adjuvant to activate NLRP3 (249). The approach has been tested *in vivo*, with the growth in B16F10-OVA melanoma and HPV E6/E7 (TC-1) tumor models inhibited by deformable nano-tumor vaccine delivery systems (250).

Although inhibition of NLRP3-associated inflammatory signaling may help limit excessive lung injury and ARDS-related inflammation, targeting this pathway in cancer presents a therapeutic dilemma. Inflammasome signaling can function as a double-edged sword with context-dependent tumor-promoting and anti-tumor effects (191). In a murine model of colitis-associated cancer, deficiency of ASC (PYCARD), caspase-1, or NLRP3 was associated with more severe colitis and increased tumor burden, supporting a protective role for inflammasome signaling in this context (251). In the same azoxymethane/dextran sodium sulfate (AOM/DSS) murine model of colitis-associated colorectal cancer, Nlrp3- or caspase-1-deficient mice developed significantly greater tumor burden (252). This effect was associated with reduced IL-18 production and consequent impairment of IFN-γ production and STAT1 activation, both of which are linked to anti-tumor activity (252). Furthermore, in a murine model of colorectal cancer liver metastasis, Nlrp3 limited metastatic growth through IL-18-dependent promotion of NK-cell maturation and tumoricidal activity, independently of IFN-γ (253).

Recent pharmacological studies further support a context-dependent anti-tumor role for NLRP3. Small molecules such as 2GBI have been shown to directly activate NLRP3, promoting inflammasome signaling and anti-tumor immunity while enhancing tumor responsiveness to immune checkpoint inhibition and reducing resistance to anti-PD-1 therapy (254). In addition, osimertinib treatment in EGFR-TKI models has been reported to induce macrophage NLRP3 activation and IL-1β release, leading to enhanced T-cell infiltration and anti-tumor immune responses (255). Similarly, Codonopsis pilosula polysaccharide suppresses NSCLC progression through NLRP3/GSDMD-dependent pyroptosis (256). In hepatocellular carcinoma, Wei et al. demonstrated that NLRP3 inflammasome components are progressively downregulated in tumor tissues as disease advances (257).This supports a potential tumor-suppressive role for NLRP3 in inflammation-driven liver carcinogenesis. Consistent with this, Dai et al. reported low NLRP3 expression in a subset of HCC cell lines (258). They further showed that NF-κB–dependent activation of the NLRP3 inflammasome inhibited HCC cell proliferation, induced G1-phase arrest, and reduced migration, which points to a tumor-suppressive role for NLRP3 in this context (258). Mechanistically, Wei et al. showed that 17β-estradiol (E2) upregulated NLRP3 inflammasome signaling in HCC cells, leading to caspase-1 activation, reduced cell viability, and increased pyroptotic cell death (259). They further demonstrated that E2 inhibited autophagy through the ERβ/AMPK/mTOR pathway, thereby promoting NLRP3-dependent caspase-1 activation and suppressing HCC progression (260). Collectively, therapeutic targeting of the NLRP3 inflammasome in cancer should be approached with caution because its effects are highly context dependent and may be either tumor-promoting or anti-tumor. Therefore, clinical translation remains challenging and will require further mechanistic, genomic, and tumor-specific studies to determine which cancer types and patient subgroups are most likely to benefit from anti-inflammasome therapy.

### 7.3 Dual therapeutic approaches

ARDS and cancer therefore share inflammatory and oxidative stress-mediated mechanisms. Hyperactivation of NF-κB, JAK/STAT, and PI3K/AKT pathways in both conditions exaggerates immune activation, endothelial malfunction, and metabolic deregulation. Both disorders show elevated levels of VEGF that cause dysfunctional angiogenesis to facilitate vascular leak in ARDS and enhanced metastasis in cancer (34, 182).

#### 7.3.1 Potential therapeutic strategies for dual targeting

Suppression of IL-6, IL-1β, and TNF-α via monoclonal antibodies or small-molecule inhibitors would diminish tumorigenesis and inflammation-induced lung damage (261). Pooladanda et al. explored the role of bromodomain 4 (BRD4) as a key epigenetic mediator connecting acute lung inflammation in ARDS to tumor metastasis. In models where ARDS was triggered by LPS, BRD4 activated the NF-κB and STAT3 pathways, leading to the release of inflammatory cytokines and increased blood vessel leakage, which helped tumors grow and spread to the lungs. BRD4 inhibition reduced inflammation and prevented lung metastases (182). These data nominate BRD4 as a translational target with the potential to address both ARDS-related lung injury and cancer metastasis. **Table 1** summarizes anti-inflammasome agents in clinical trials for diverse indications, which may be candidates for repurposing against ARDS and cancer.

**Table 1:** Clinical agents targeting the inflammasome–IL-1/IL-6 axis (and upstream modulators) with relevance to ARDS and cancer: mechanism, repurposing rationale, and representative clinical trials.

Drug/Agent	Mechanism	ARDS- or cancer-related indication	Key clinical trial (NCT)	Route of administration	Target population	Ref.
Anakinra (IL-1 receptor antagonist)	Blocks IL-1 receptor 1→ ↓ IL-1α &β signaling	Regulates IL-1–induced hyperinflammation; assessed in severe pneumonia/ARDS and investigated for tumor-promoting inflammation (including early ICU, non-COVID ARDS).	NCT04341584	SC (standard); IV (ICU, off label when rapid effect/poor SC absorption)	Adults hospitalized with pneumonia/ARDS When early ICU non-COVID ARDS (should meet strict trial protocol)	(10, 262–264)
Canakinumab (anti–IL-1β mAb)	Neutralizes IL-1β→ ↓ fever, CRP, WBCs recruitment etc.	CANTOS: ↓ recurrent CV events in prior MI with hsCRP ≥2 mg/L; exploratory ↓ incident lung cancer (dose signal). NSCLC therapy trials (CANOPY-1/-2): negative	NCT01327846	Subcutaneous (q3 months)	Prior MI with hsCRP ≥2 mg/L	(265–268)
Colchicine (microtubule inhibitor/indirect NLRP3 modulator)	Inhibits microtubule polymerization → dampens neutrophil trafficking/activation and can reduce NLRP3 inflammasome assembly/IL-1β signaling (indirect)	NLRP3/neutrophil-driven lung inflammation modulator, COVID-19 outpatient RCTs show mixed benefit (COLCORONA signal; ACT negative), hospitalized COVID-19 negative (RECOVERY), preclinical ALI/ARDS models suggest reduced neutrophilic inflammation	NCT04322682	Oral	Outpatients ≥40 years with COVID-19 and risk factors	(269–273)
Dapansutrile (OLT1177) (oral NLRP3 inhibitor)	Selective NLRP3 blockade → ↓ caspase-1 activation → ↓ maturation/secretion of IL-1β & IL-18; ↓ pyroptosis	Direct NLRP3 inflammasome inhibition. Early human signals in acute gout and HFrEF; Development expanding to cardiometabolic and neuroinflammatory indications	NCT05658575	Oral	Acute gout flares; exploratory cardiovascular inflammation.	(274–277)
N-Acetylcysteine (NAC)	Cysteine donor/antioxidant → ↑ (GSH), ↓ ROS → may attenuate redox-dependent NLRP3 priming	Evaluated as adjunct therapy in ALI/ARDS and COVID-ARDS, but human data are inconsistent (heterogeneous timing, route, dose; small/variable trials)	NCT05706402	Oral	Adults with early ARDS Adults hospitalized with an acute exacerbation of COPD	(278–282)
Rosuvastatin (Statin with pleiotropic anti-inflammatory effects)	Statin—blocks HMG-CoA reductase (↓ mevalonate → ↓ LDL-C) ↓isoprenoid-dependent signaling (Rho/Rac) → anti-inflammatory and endothelial-stabilizing effects	Sepsis-ARDS (SAILS), no clinical improvement, arguing against routine statin use for established ARDS.	NCT00979121	Oral	Adults with sepsis-induced ARDS requiring mechanical ventilation	(283, 284)
Tocilizumab (IL-6R mAb)	Blocks membrane & soluble IL-6receptor signaling → attenuates JAK/STAT-mediated inflammation	Severe COVID-19 pneumonia (REMAP-CAP/RECOVERY): ↓ 28-day mortality when added to steroids	NCT02735707	Intravenous	Critically ill adults requiring organ support	(285, 286)

ALI, acute lung injury; ARDS, acute respiratory distress syndrome; ASCVD, atherosclerotic cardiovascular disease; CAPS, cryopyrin-associated periodic syndrome(s); COPD, chronic obstructive pulmonary disease; COVID-19, coronavirus disease 2019; CRP, C-reactive protein; CRS, cytokine release syndrome; CV, cardiovascular; GSH, reduced glutathione; HFrEF, heart failure with reduced ejection fraction; hsCRP, high-sensitivity C-reactive protein; ICU, intensive care unit; IL, interleukin; IL-6R, interleukin-6 receptor; IV, intravenous; JAK/STAT, Janus kinase/signal transducer and activator of transcription; mAb, monoclonal antibody; MI, myocardial infarction; NAC, N-acetylcysteine; NCT, ClinicalTrials.gov identifier; NLRP3, NOD-, LRR- and pyrin domain-containing protein 3; NSCLC, non-small-cell lung cancer; RA, rheumatoid arthritis; ROS, reactive oxygen species; SC, subcutaneous; sJIA, systemic juvenile idiopathic arthritis; SAILS, Statins for Acutely Injured Lungs from Sepsis trial; WBCs, white blood cells.

Symbols: ↑ increase; ↓ decrease; ≥ greater than or equal to.

**Table 1**. This table includes agents that target the NLRP3 inflammasome either directly or indirectly through modulation of downstream inflammatory mediators. In current clinical practice, most available therapies act indirectly, particularly by targeting cytokines such as IL-1β, rather than by directly inhibiting NLRP3 itself. Given that both ARDS and cancer are driven, at least in part, by dysregulated inflammatory signaling, repurposing therapies that modulate this pathway may have translational value, especially in settings where one conndition may predispose to or complicate the other. However, such strategies warrant careful consideration because interference with inflammasome signaling may also affect host defense and anti-tumor immunity.

A recently published study further supports the potential for targeting NLRP3 across both acute and chronic inflammatory conditions (287). Specifically, nimbolide was shown to ameliorate LPS-induced ARDS and DSS-induced chronic colitis, the latter being a condition that can predispose to colitis-associated colorectal cancer (287). Although this does not directly establish a causal ARDS-cancer relationship, it reinforces the concept that NLRP3 may represent a shared and temporally relevant therapeutic target across distinct inflammatory disease settings. These findings are consistent with our hypothesis-generting framework and highlight the need for further studies exploring whether similar cross-disease targeting strategies may be relevant at the ARDS–cancer interface.

## 8 Future perspectives

The preceding discussion highlights several key avenues for future research. First, the role of other inflammasomes (beyond NLRP3) in both diseases requires clarification, and those inflammasomes reported to be involved in ARDS, such as NLRC4 and AIM2, merit further investigation in cancer. Second, investigating the role of comorbidities in shaping inflammasome responses could help to explain heterogeneous clinical outcomes as well as how best to tailor anti-inflammatory strategies. There is still a need to understand how NLRP3 inflammasomes interact with immune pathways implicated in both ARDS and cancer, such as RIG-I-like receptors (RLRs), stimulator of interferon genes (STING), and TLRs, as all are considered promising cancer immunotherapy targets (288). In addition, the discovery of new and advanced biomarkers will assist in providing more precise understanding of the pathophysiology and the molecular predisposition of the disease. For example, the use of multi-omics has been successfully used to discover biomarkers and reveal mechanisms involved in inflammatory lung diseases (289), so using multi-omics approaches to integrate data from different biological layers such as epigenomics, metabolomics, proteomics, transcriptomics, and genomics would be a promising means to offer a systems-level view of the activation of NLRP3 inflammasomes to discover effective targets for future treatments. Furthermore, advanced *in vitro* models such as tissue chip technology have shown promise in the pharmaceutical industry and for drug discovery, as they can accurately emulate human physiology and enhance the predictive power of preclinical models. Tissue chip technology increases understanding of the diseases and enables the discovery of toxicity and disease-related biomarkers (290). These advanced models can further be utilized to design patient-centered, disease-specific medicines that not only to control symptoms but also modify disease progression in affected patients (290).

## 9 Conclusion

The interplay between ARDS and cancer in the context of inflammasome pathways represents an intricate yet provocative area of research. While ARDS can be a significant life-threatening condition that can arise secondary to cancer, there is insufficient literature dedicated to the relationship between the two conditions. The systemic effects of ARDS and its shared pathways with cancer suggest new areas for exploring disease pathogenesis and treatment approaches. Inflammasomes, especially NLRP3, are among the main players in this process. The role of other inflammasomes implicated in this process require further investigation, utilizing advanced strategies and disease models for precise biomarker discovery and mechanistic understanding. Through further elucidation of these connections, healthcare professionals can tailor effective, personalized treatments for both ARDS and cancer, which both lack curative treatments.
